# Step-by-step teaching method: improving learning outcomes of undergraduate dental students in layering techniques for direct composite resin restorations

**DOI:** 10.1186/s12909-020-02230-1

**Published:** 2020-09-11

**Authors:** Jia-xue Yuan, Ke-yu Yang, Jing Ma, Zhen-zhen Wang, Qing-yu Guo, Fei Liu

**Affiliations:** 1 Clinical Research Center of Shaanxi Province for Dental and Maxillofacial Diseases, College of Stomatology, Xi’an Jiaotong University, Xi’an, 710004 PR China; 2 Department of Pediatric Dentistry, Affiliated Stomatology Hospital of Xi’an Jiaotong University, Xi Wu Road No.98, Xi’an, 710004 PR China; 3 Department of Endodontics, Affiliated Stomatology Hospital of Xi’an Jiaotong University, Xi’an, 710004 PR China; 4 Department of Prosthodontics, Affiliated Stomatology Hospital of Xi’an Jiaotong University, Xi’an, 710004 PR China

**Keywords:** Teaching methods, Dental restoration repair, Tooth injuries, Composite resins

## Abstract

**Background:**

Layering techniques for direct composite resin restorations might be complicated for inexperienced learners, as a number of materials and instruments are required at each step. The present study aimed to compare and assess the teaching effect of step-by-step and all-in-one teaching methods in layering techniques for direct composite resin restorations among undergraduate dental students.

**Methods:**

A total of 68 junior dental students participated in this study, which was a prospective and single-blind trial. The students were randomly divided into a step-by-step group (experimental group, *n* = 34) and all-in-one group (control group, *n* = 34). The same teacher taught the two groups, ensuring a comparable teaching effect. The final score of each student was an average of scores by two experts who were blinded to the grouping. The scoring system was consisted by five parts. Each part was assigned scores of 3.0, 1.5, or 0. The total maximum score was 15 and minimum was 0. The total time taken by each group was also calculated.

**Results:**

The values of the quality of tooth restorations evaluated by experts for step-by-step and all-in-one groups were 11.29 ± 2.13 from 15 and 9.00 ± 2.71 from 15 (t = 3.88, *P* < 0.001), respectively. In addition, the time spent by the experimental group was significantly lesser than that spent by the control group, which was 122.47 ± 2.82 and 137.18 ± 6.75 min, respectively (t = 11.72, *p* < 0.001).

**Conclusion:**

With regard to the layering techniques for direct composite resin restorations, the outcomes were better in the step-by-step group than in the all-in-one group.

## Background

Traumatic dental injuries (TDIs), which are increasingly becoming a public health problem [1], are collision injuries involving the anterior region of the mouth. TDIs are more prevalent in children and adolescent, and mainly involve the maxillary central incisors in young permanent teeth [2]. Dental caries and dental abnormalities are other common causes of defects in young permanent teeth [3]. These defects in young permanent teeth, particularly in the anterior region, might cause significant esthetic, functional, and psychosocial problems in children, and often require attention [4]. Appropriate treatment and restoration of defective anterior teeth are important [5]. Direct restoration with resin and reattachment of fractured tooth fragment are economical and efficient approaches to treat fractured teeth [5, 6]. However, in most of the cases, restoration with resin composites is preferred. For example, when the fracture has not caused excessive tooth loss, or when the defect is caused by dental caries in young permanent teeth, restoration with resin composites is the first choice of both patients and dentists [5, 7, 8].

Resin composites are suitable for young permanent teeth as they preserve most of the healthy tooth structure [9]. With the advancement in technology, it has become convenient to imitate the natural tooth structure and morphology using resin composites, in particular, the layering techniques for direct composite resin restorations [10, 11]. However, performing these procedures requires not only the knowledge of dental anatomy, but also the manual skills to achieve ideal outcomes, which is a challenge for the operators [12].

Unlike other disciplines, dentistry is more practical in nature. Manual skill training is an important aspect of dental students’ education. The normative course of layering techniques for direct composite resin restorations comprised theoretical lectures, and practical demonstrations by teachers, followed by learners’ skill practice. In the traditional teaching course, all these three tasks (theoretical lecture, practical demonstration, and skill practice) were performed one after another, which was called the all-in-one teaching method. However, the teaching effectiveness of the all-in-one method was not quite satisfactory, especially when a procedure involved multiple steps. For example, the layering techniques for direct composite resin restorations comprises eight steps: (1) wax pattern preparation, (2) silicone rubber model preparation, (3) shade selection, (4) beveled preparation, (5) acid etching and bonding, (6) silicon palatal guide to control the palatal contours and restore the incisal edge, (7) layering technique to build up the restoration, and (8) polishing. Though students were taught and had practiced some of these procedures in other specialized courses (for example, pattern preparation in Oral Anatomy and Physiology, impression preparation in Prosthetic Dentistry, and use of composite resin in Endodontic and Operative Dentistry), they might still be difficult for inexperienced learners as each procedure requires the use of a number of materials and instruments. Consequently, an efficient teaching method is necessary.

Recently, an innovative teaching method called the step-by-step method has been applied in dental education. To our knowledge, there are a few articles about the step-by-step method in dental education, and these articles focused on crown preparation. The participants were dental residents [13] and third- and fourth-year dental students [14]. However, the application of the step-by-step method in other dental courses, such as pediatric dentistry or operative dentistry, has not been studied.

The aim of the present study was to assess the teaching effectiveness of the step-by-step method in layering techniques for direct composite resin restorations among undergraduate dental students, which could be a dependable alternative for dental manual skill training.

## Methods

This study was a prospective trial involving third-year undergraduate dental students from the college of stomatology of Xi’an Jiaotong University Health Science Center. As the course of layering techniques for direct composite resin restorations is compulsory for third-year dental students, all 68 students in the third year participated in the present study. They were randomly divided into two groups: step-by-step (experimental group, *n* = 34) and all-in-one (control group, *n* = 34). The students attended the afternoon class for 2 days, consecutively. The same teacher taught the two groups, ensuring a comparable teaching effect. All the students were informed beforehand that the course content was same in the two groups, while the curriculum structure was different. The all-in-one group was taught the first day using the traditional teaching method, while the step-by-step group was taught the next day. This arrangement ensured, as much as was possible, that there was no communication about the new teaching method among the students. Approved by the medical ethics committee of the affiliated Stomatology Hospital of Xi’an Jiaotong University, this study was determined to be a regular pedagogical practice, and no human subject research or personal information of participants was used in the study (xjkqll2017–019). Verbal consent was obtained from the students before the study.

**Fig. 1:**
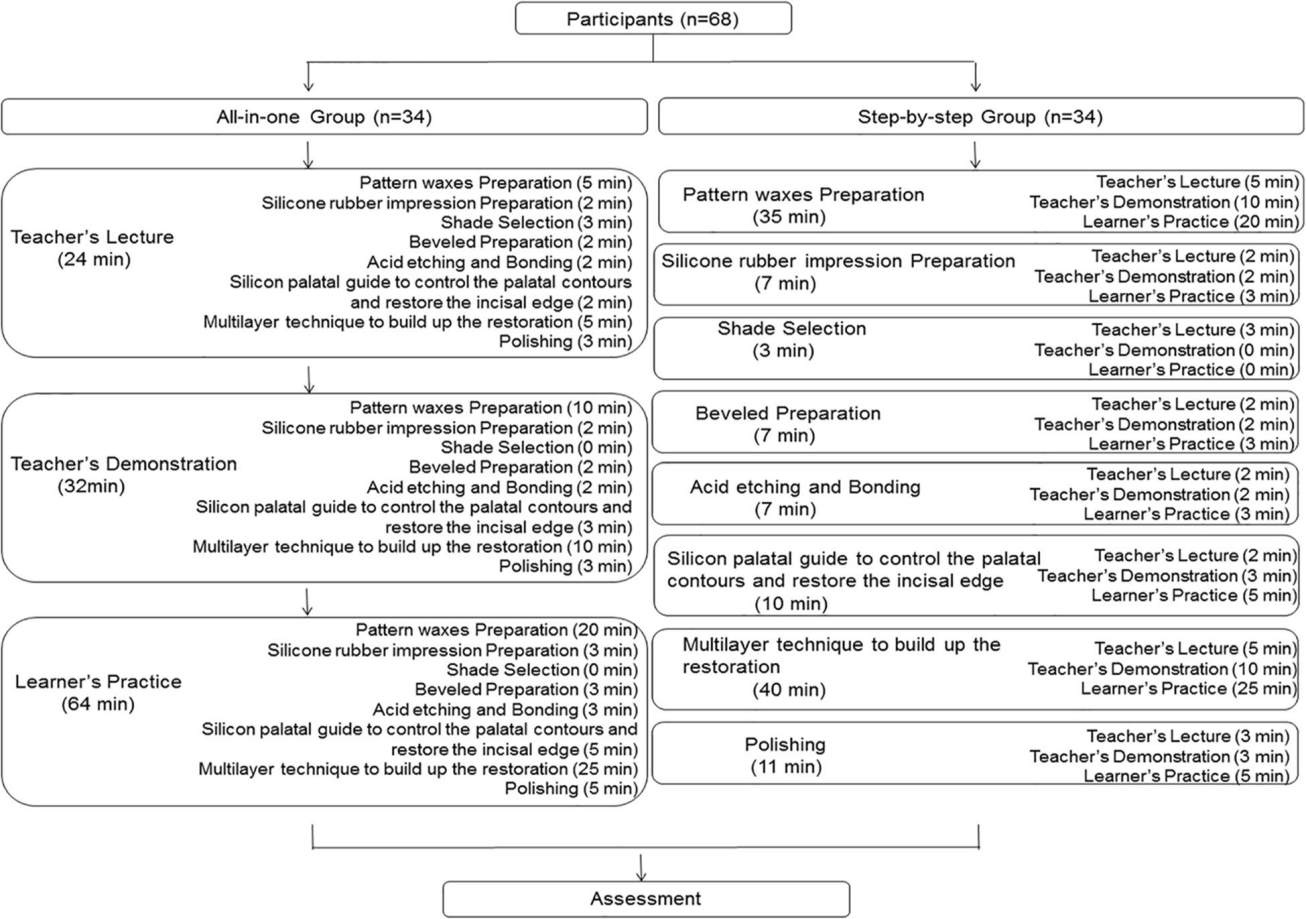
Structure of the courses of the all-in-one group and step-by-step group

We prepared plastic anterior teeth to simulate dental trauma on young permanent teeth, with nearly half of the crown having defective incisal angles and marginal ridges. During the class, the learners restored the anterior teeth using layering techniques for direct composite resin restorations. For the control group, the teacher first gave a 24-min theoretical lecture, followed by a 32-min practical demonstration of skills required for layering techniques and direct composite resin restorations. Then, the students were given 64 min for skill practice. For the experimental group, the 2-h class was segmented into eight parts as per the steps mentioned earlier. Time allocation for each part is shown in Fig. 1. The students were allowed to ask questions during the practice time. Two teaching assistants also walked around the classroom to help students when they encountered any problem. For shade selection, only a theoretical lecture was reserved. After completion of the class, the quality of tooth restorations was evaluated by two independent experts with more than 5 years of clinical experience who had majored in endodontics and prosthetics, respectively. Each part was assigned scores of 3.0, 1.5, or 0. The total maximum score was 15 and minimum was 0. The higher the score, the better the result. The detailed standard for evaluation is shown in Table 1. The evaluation scale consisted of the anatomic morphology of the palatal surface, labial surface, and incisal edge; quality of silicone rubber model; and degree of polishing. The scoring was based on a traditional system used in our hospital for more than 5 years, which was discussed and revised by the teaching group and specialists. The scorers were blinded to the grouping. The final score of each student was an average of the scores given by the two experts. The total time allowed was the same for both groups, while the actual time taken by each group was also calculated.

**Table 1:** Parameters followed in layering techniques and direct composite resin restorations

Parameter	Grades		
	3 points	1.5 points	0 points
part 1	Optimal palatal anatomic morphology	Moderately palatal anatomic morphology	No palatal anatomic morphology
part 2	Optimal resettability and clear edge	Moderately resettability but a few bubbles that do not affect the use	Unable to reset or a lot of bubbles that need to be remodeled
part 3	Optimal clear layer and mamelon	Optimal clear layer or mamelon	No clear layer or mamelon
part 4	Optimal labial surface anatomic morphology	Moderately labial surface anatomic morphology	No labial surface anatomic morphology
part 5	Optimal degree of finish	Moderately degree of finish	Poor degree of finish or the presence of overhang

The data were recorded and analyzed using the Statistical Package for the Social Sciences (IBM, SPSS version 13.0, IL, USA). Independent samples t-tests were conducted for analyzing the age of participants, quality of tooth restorations, and the time spent by the two groups. The chi-squared test was conducted for analyzing the gender of participants, with the significance level set at *p* < 0.05.

## Results

The teaching effectiveness of the all-in-one and step-by-step methods in layering techniques for direct composite resin restorations of anterior teeth was compared. Sixty-eight dental students from college of stomatology of Xi’an Jiaotong university health science center participated in this study. The main characteristics of all the students were homogeneous at baseline. There were 19 female and 13 male participants in the all-in-one group, and 21 female and 15 male participants in the step-by-step group, with no significant difference between the two groups (χ^2^ = 0.008, *p* > 0.05). The average age of students in the all-in-one and step-by-step groups was 21.26 ± 1.02 and 21.23 ± 1.26, respectively, with no significant difference (t = 0.106, *p* > 0.05).

The value of quality of tooth restorations evaluated by the experts for the step-by-step and all-in-one groups was 11.29 ± 2.13 and 9.00 ± 2.71 (t = 3.88, *p* < 0.001), respectively. In terms of the decomposed evaluation index, there were significant differences between the control group and the experimental group for parts 1 (t = 3.78, p < 0.001), 4 (t = 4.15, *p* < 0.001), and 5 (t = 2.91, *p* < 0.01) (Table 2). In addition, the actual time spent by the experimental group was significantly less than that spent by the control group, which was 122.47 ± 2.82 and 137.18 ± 6.75 min, respectively (t = 11.72, *p* < 0.001). Overall, better outcomes were obtained in the experimental group regarding the layering techniques for direct composite resin restorations of anterior teeth.

**Table 2:** Learner’s achievement in the all-in-one group and step-by-step group

Parameter	Assessment			
	All-in-one	step-by-step	t	*P*
part 1	1.63 ± 1.07	2.47 ± 0.73	3.78	< 0.001
part 2	2.25 ± 0.99	1.98 ± 0.96	1.12	> 0.05
part 3	1.85 ± 0.98	2.16 ± 0.84	1.39	> 0.05
part 4	1.50 ± 0.94	2.34 ± 0.76	4.15	< 0.001
part 5	1.76 ± 0.78	2.34 ± 0.84	2.91	< 0.01
Total	9.00 ± 2.71	11.29 ± 2.13	3.88	< 0.001

## Discussion

TDIs, dental caries, and dental abnormalities are frequently occurring diseases dealt by the department of pediatric dentistry; they commonly involve the maxillary central incisors in young permanent teeth. Because of the presence of special anatomic morphology features in young permanent incisors, such as mamelons and developmental grooves, the rehabilitation of young permanent incisors is different and more difficult than that of permanent teeth [15]. The operator’s manual skills and familiarity with the procedure, knowledge of dental anatomy, and restorative materials are important factors for success. This study aimed to evaluate the effectiveness of different teaching methods regarding layering techniques for direct composite resin restorations, and found that better outcomes were obtained using the step-by-step teaching method.

This result is in line with the findings of previous studies, which showed that the step-by-step method results in better teaching effectiveness in crown preparation. Liu et al. found that dental residents in the step-by-step group performed better than those in the all-in-one group in crown preparation, regardless of whether the outcome was evaluated by learners, experts, or digital systems [13]. Lukas et al. [14] combined the multimedia approach with the step-by-step method in the teaching of crown preparation, and found that 94% of the undergraduate dental students responded favorably to this method, while the teaching faculty felt that this method of instruction increased efficiency. The better outcomes of the step-by-step method can be attributed to several factors. First, this teaching method put forward more requirements for teachers. Teachers need to refine every step of the procedure and make it as detailed as possible, which might be an essential factor to ensure and improve the teaching quality. Second, it changes the teacher-centered or student-centered approach to teacher-student-interactive approach [16]. We observed that in classrooms where the step-by-step method was adopted, there was a more close and positive interaction between students and teachers. Frequent interactions benefit students as they perform better and achieve their goals in class. Third, it can make the teaching process highly efficient. In the present study, the total time spent by the step-by-step group was significantly lesser than that spent by the all-in-one group since the teaching process was easier to control.

In addition, the step-by-step method decreases the cumulative effect of errors in each step and avoids irreparable errors in the final outcome [13]. According to the evaluation indexes, the all-in-one group preformed less satisfactorily than the step-by-step group in three parts. The first part was preparing the wax pattern, which is a basic model for restoration. The quality of the wax pattern determines the shape and anatomy of the restoration. Cumulative minor errors in this part may cause performance issues in the subsequent parts, including labial surface morphology and polishing. More careful observation and enhanced short-term memory were the favorable factors that helped students in the step-by-step group reduce errors. In the all-in-one group, students would practice by themselves after nearly 1 h of theoretical lecture and practical demonstration. In contrast, the interval was 5–15 min in the step-by-step group, allowing the students to put into practice what they have heard and seen, thus making the teaching procedure highly efficient.

With advances in technology, traditional teaching methods are confronted with the challenge of innovative teaching methods, such as video- or net-based teaching [17]. As dentistry is a practical discipline, pre-clinical manual skills training courses play an important role in dental education. However, video- or Internet-based teaching has limitations in that there is a loss of physical contact between teachers and students, and timely guidance is not always available. Thus, hands-on courses involving many senses facilitate the improvement of dental students [18].

### Limitations

As the first trial of the step-by-step teaching method in pediatric dentistry, we found better outcomes with the step-by-step teaching method. However, there are some limitations to this study. First, the results should be interpreted with caution because the sample size and time of follow-up was finite. Second, the general applicability of the step-by-step teaching method remains unknown. A complex multi-step layering technique for direct composite resin restorations was the only technique applied in the present study. The outcomes for other techniques, in particular, simple techniques such as pit and fissure sealing or preventive resin restoration, should be studied in the future. Third, two experts from different departments scored the restorations with different considerations. Although the final score of each student was averaged, no calibration was done for the examiners, which might have resulted in some potential bias.

## Conclusion

In conclusion, the step-by-step teaching method provides better outcomes in layering techniques for direct composite resin restorations among undergraduate dental students. As it facilitates a more efficient teaching process; close and positive interaction between students and teachers; and results in less cumulative effect of errors in each step, the step-by-step teaching method might be effective in dental skills training. However, similar studies with larger sample sizes, multiple centers, and a larger range of dental skills are required to generalize the results.

## Data Availability

The datasets used and/or analyzed during the current study are available from the corresponding author on reasonable request.

## Abbreviations

TDIs: Traumatic dental injuries
